# From screens to cognition: A scoping review of the impact of screen time on cognitive function in midlife and older adults

**DOI:** 10.1177/20552076251343989

**Published:** 2025-07-10

**Authors:** Rinanda Shaleha, Nelson Roque

**Affiliations:** 1 Department of Human Development and Family Studies, The Pennsylvania State University, University Park, PA, USA; 2 Center for Healthy Aging, The Pennsylvania State University, University Park, PA, USA

**Keywords:** Screen time, screen use, cognitive function, aging population, digital technology

## Abstract

**Background:**

With increasing digital engagement across the lifespan, understanding how screen use influences cognitive aging is a growing public health priority. While much research has focused on younger populations, the cognitive implications of screen time in midlife and older adulthood remain underexplored. This scoping review synthesizes recent evidence on the associations between screen-based behaviors and cognitive outcomes in adults aged 40 and older.

**Methods:**

Following PRISMA-ScR guidelines, we systematically searched PubMed and CINAHL for studies published between 2018 and 2023. Eligible studies examined the relationship between screen use and cognitive function in adults aged 40 and above. Data were charted across screen types, cognitive domains, and study characteristics.

**Results:**

Seventeen studies met the inclusion criteria. Active screen use was generally associated with better cognitive outcomes, particularly in memory, executive function, and attention. In contrast, passive screen use was linked to poorer outcomes, including verbal memory and global cognition declines. Findings varied across cognitive domains and were influenced by moderators such as education, physical activity, sleep quality, and digital attitudes.

**Conclusions:**

Screen time during midlife and older adulthood carries both risks and potential cognitive benefits. Its effects are specific to different domains and depend on context. Future research should adopt more nuanced, longitudinal, and inclusive approaches to guide digital health strategies that promote cognitive well-being in aging populations.

## Introduction

As global life expectancy rises and populations age, promoting cognitive health has become a public health priority.^1,2^ Concurrently, digital technology has become deeply embedded in daily life, reshaping how individuals access information, communicate, and manage everyday activities.^3^ Digital engagement provides valuable opportunities for cognitive stimulation, skill-building, and maintaining independence in midlife and older adulthood, benefiting both cognitively healthy individuals and those experiencing mild cognitive impairment.^4,5^ For instance, active use of digital technologies can foster continuous learning, curiosity, and social connectivity, which are critical factors supporting cognitive resilience and healthy aging.^6,7^

Despite these potential advantages, midlife and older adults remain understudied in screen-time research compared to younger populations. Given the distinct cognitive, social, and technological contexts of older adults, findings from youth-oriented studies may not directly apply. Moreover, digital engagement among older adults also carries risks, including attention deficits, disrupted sleep patterns, social isolation, reduced physical activity, and structural brain changes that could accelerate cognitive decline and dementia onset.^6,8,9^ Extended screen exposure may further contribute to visual fatigue, chronic distraction, and sedentary behaviors, potentially exacerbating cognitive vulnerabilities.^10,11^

Given these complexities, understanding the nuanced relationship between digital screen time and cognitive function in aging populations is crucial. This scoping review systematically synthesizes recent empirical research (2018–2023) on the associations between screen time and cognitive outcomes in midlife and older adults. By integrating findings across diverse cognitive domains, device types, and usage contexts, this review identifies cognitive risk and benefit patterns and provides clear direction for future research and intervention development.

## Methods

### Search strategy and information sources

We conducted a comprehensive literature search using two primary databases: PubMed and CINAHL. These platforms were selected to ensure broad coverage of peer-reviewed studies in biomedical, cognitive, and public health research. We included PubMed to provide comprehensive access to research in cognitive aging and neurological outcomes from a medical perspective. We acknowledge this may have limited the inclusion of some psychology-focused studies and address this limitation in the Discussion.

The search was conducted for literature published between 2018 and 2023 to capture the most recent evidence during a period of increased digital technology integration, particularly post-COVID-19. The search string was: (“Screen time”[MeSH Terms] OR “Screen use” OR “Digital screen exposure” OR “Computer use” OR “Television watching” OR “smartphone use”[All Fields]) AND (“Cognitive performance” OR “Cognitive function” OR “Cognitive decline” OR “Cognitive impairment” OR “Aging and cognition”). Although the term “tablet” was not explicitly included in the search strategy, several included studies did examine tablet use. These were likely captured through broader terms such as “screen use” and “digital screen exposure,” which conceptually encompass a range of digital devices, including tablets.

### Eligibility criteria

Studies were eligible for inclusion if they were peer-reviewed empirical articles, published in English between 2018 and 2023, and focused on adults aged 40 years or older. Eligible studies examined screen time or screen-based device use, such as television viewing, computer use, or smartphone use, and reported outcomes related to cognitive function, including measures of performance or cognitive decline. We excluded review articles, theoretical papers, book chapters, dissertations, and conference abstracts without full text. Studies that did not include cognitive outcomes or focused exclusively on children, adolescents, or young adults were also excluded.

### Selection of sources of evidence

**Figure 1.**
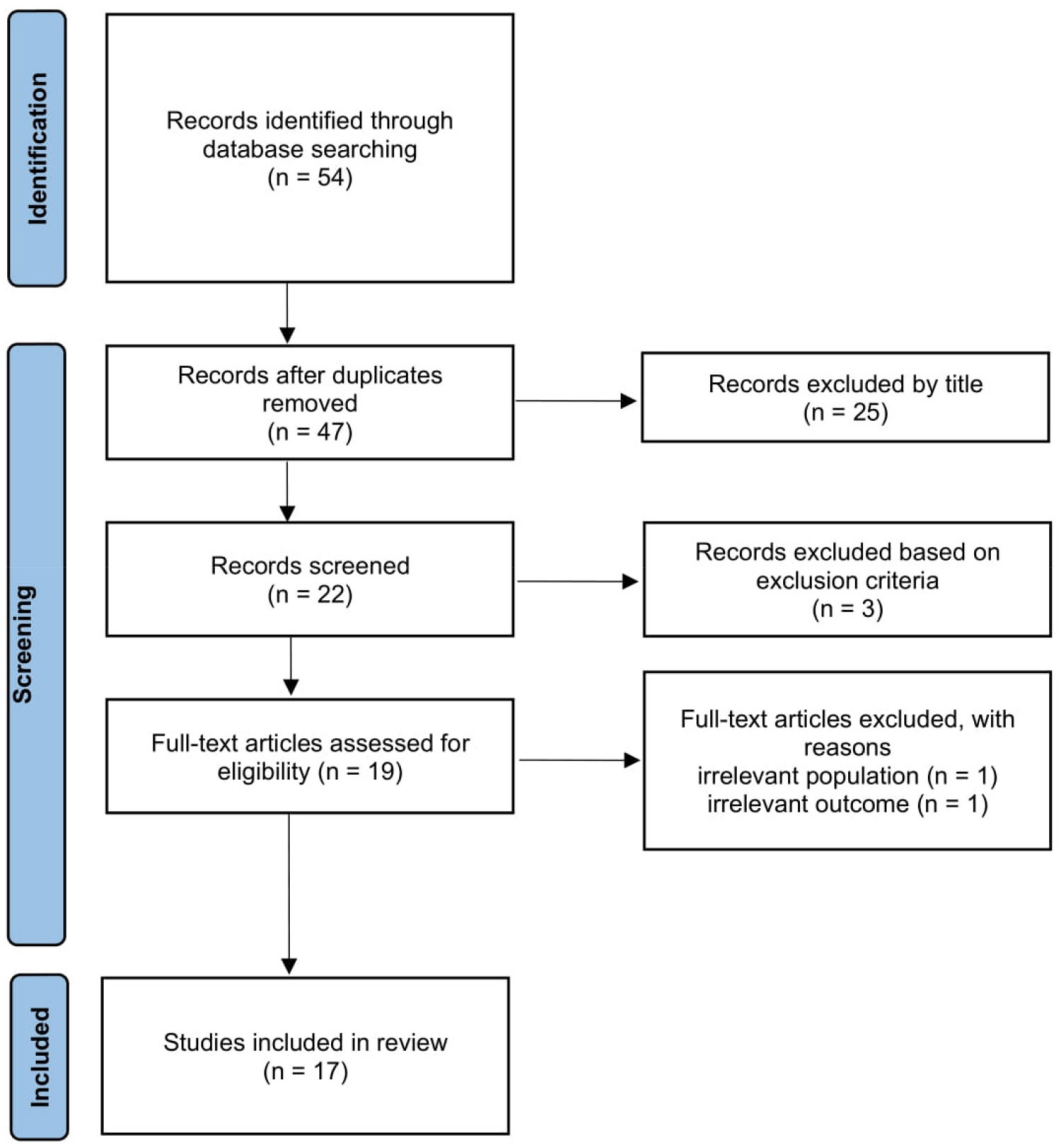
Study selection flow chart (PRISMA-ScR flow diagram).

The initial search yielded 54 records. After removing duplicates, 47 records remained. Titles and abstracts were screened, resulting in the exclusion of 25 records that did not meet the basic inclusion criteria. The remaining 22 records underwent abstract-level screening, of which 3 were excluded based on more specific exclusion criteria. This left 19 full-text articles that were assessed for eligibility. Following full-text review, two articles were excluded due to having an irrelevant population (*n* = 1) or outcome (*n* = 1). Ultimately, 17 studies were included in the final synthesis. The screening and selection process is summarized in Figure 1 (PRISMA-ScR flow diagram).

### Data charting process

Data from the included studies were charted systematically using a predefined matrix table. The matrix table (see Appendix A) was designed to ensure consistency and comprehensiveness in data extraction, covering key variables such as the author's first name and year of publication, study location, study type, purpose or aims of the study, participant demographics (including sample size) and screen use, cognitive performance measures, and key findings or discussions. The matrix table was reviewed and tested by the authors to confirm that it effectively captured all necessary information before being applied across all included studies. To enhance the reliability of the data extraction process, two reviewers independently charted the data. Any discrepancies between the reviewers were resolved through discussion. Where data was unclear or incomplete, corresponding authors were contacted to obtain additional information and confirm the accuracy of the extracted data.

### Synthesis of results and approach to evidence

The data extracted from the 17 included studies were synthesized using a narrative approach. In keeping with the scoping review methodology, we synthesized the findings using a narrative and thematic approach. This allowed us to map the breadth of existing research and identify patterns in how different forms of screen use were associated with cognitive outcomes in midlife and older adults. The synthesis focused on identifying patterns and themes within the data, particularly regarding how different types of screen use (e.g. television watching, smartphone use, computer use) influenced various cognitive domains, such as memory, attention, and executive function.

To provide descriptive insight into the methodological quality of the included studies, we adapted elements from the Mixed Methods Appraisal Tool (MMAT), a framework designed to assess the quality of qualitative, quantitative, and mixed-methods research.^12^ We used it to guide our evaluation. These considerations informed our data charting in Appendix A and are referenced throughout the Results and Discussion. This approach offers readers a contextual understanding of the evidence bases while maintaining the inclusive nature of a scoping review.

## Results

### Overview of included studies

#### Study characteristics

After removing duplicates, 47 unique records remained from an initial pool of 54. Following title, abstract, and full-text screening based on predefined eligibility criteria, 17 studies were included in the final scoping review (Figure 1). These studies included over 27,000 participants, with sample sizes ranging from 32 to 5356. Most participants were midlife and older adults, with some studies focusing on cognitively healthy individuals and others including those with mild cognitive impairment or early neurological conditions. Full details on participant characteristics and study design are provided in Appendix A.

In terms of study design, seven studies were cross-sectional,^13–19^ and nine studies employed longitudinal designs.^20–28^ One study^29^ employed a qualitative Delphi expert consensus mapping approach to identify behavioral indicators of cognitive decline, while another^15^ focused on psychosocial predictors of screen-related behavior without formal cognitive testing.

Geographically, the studies included in this review were concentrated in high-income Western countries but also reflected growing contributions from parts of Asia and Latin America. Seven studies were conducted in the United States.^13,14,16,23,24,25,26^ There were two from the United Kingdom,^27,29^ one from Belgium,^15^ and one from Italy.^17^ Asian representations included four studies: 1 from mainland China,^20^ 1 from Taiwan,^21^ 1 from Japan,^22^ and 1 from India.^18^ One study was conducted in Brazil, representing South America^19^ Notably, no studies were from Africa, Oceania, Eastern Europe, or the Middle East. This geographic skew limits the generalizability of findings. Expanding research efforts to underrepresented regions will be essential for building a more comprehensive understanding of how digital screen use relates to cognition across cultural and socioeconomic contexts.

#### Devices and purposes

Studies assessed four main types of screen-based devices: television, computers (desktop or laptop), smartphones, and tablets. The types of devices examined in each study are summarized in Table 1. Computers were the most frequently examined (13 of 17 studies),^13,14,16,17,19,20,22–27,29^ followed by televisions (6 studies),^15,16,19–21,28^ smartphones and cell phone (4 studies),^17–20^ and tablets (3 studies).^16,17,19^ Although tablets were not explicitly included in the search terms, they appeared in two studies, suggesting that broader keywords like “screen use” effectively captured diverse devices.

**Table 1.** The cognitive domain measured in the included studies.

Year	Author (year)	Screen type	Cognitive domain(s)	Test(s) used
2019	Calhoun & Lee	Computer	Fluid intelligence (Verbal Reasoning)	Verbal Analogies Test (Verbal series score)
	Couth et al.	Computer (desktop/laptop)	Memory, Executive Function, Language	Expert consensus mapping (Delphi method); no formal cognitive battery used
	Fancourt & Steptoe	TV	Verbal memory, Semantic fluency	The Battery of Neuropsychological Tests CERAD; Verbal Memory Task (Immediate + Delayed Word Recall); Semantic Fluency (Animal Naming—60 s task)
	Krell-Roesch et al.	Computer	Memory, language, attention/executive function, visuospatial	Auditory Verbal Learning Test, Wechsler Memory Scale; Boston Naming Test; Wechsler Adult Intelligence Scale; Trail Making Test Part B; Digit Symbol Substitution Test
	Mackenbach et al.	TV	None (N/A)^a^	Self-report survey (TV viewing, attitudes, modeling)
2020	Nori et al.	Computer	Fluid intelligence (Nonverbal reasoning), Crystallized intelligence (Verbal knowledge)	Kaufman Brief Intelligence Test-2 (KBIT-2): Verbal IQ (VIQ = Crystallized), Nonverbal IQ (NVIQ = Fluid)
2021	Bernstein et al.	Passively monitored home computer use and application use (e.g. email, browser, word processing, games)	Global cognition, memory, language, executive function, attention, visuospatial construction	Trail Making Test Part A & B; Stroop Color Naming, Stroop Word Reading, Stroop Color-Word Interference, Number Span Forward & Backward, Craft Story Immediate and Delayed Recall, Consortium to Establish a Registry for Alzheimer's Disease (CERAD) Word List Delayed Recall and Recognition, Benson Complex Figure Copy and Delayed Recall, Multilingual Naming Test (MINT), Category Fluency
	Kurita et al.	Computer (word processing, email, internet, games)	Memory, attention, executive function, processing speed	The National Center for Geriatrics and Gerontology-functional Assessment Tool (NCGG-FAT): Word List Memory I (Immediate Recognition); Word List Memory II (Delayed Recall); Trail Making Test A & B; Symbol Digit Substitution Task
	Tuteja et al.	Smartphone	Global cognition, Memory, Attention, Visuospatial ability, Language, Executive function	Montreal Cognitive Assessment (MoCA)
2022	Bernstein et al.	Computer, wearable sleep tracker, instrumented pillbox	Global cognition, Memory, Executive function, Language, Attention, Visuospatial construction	Trail Making Test Part A & B; Stroop Color Naming & Color-Word Interference; Number Span Forward & Backward; Craft Story Immediate Recall, Craft Story Delayed Recall, CERAD; Word List Delayed Recall; Benson Complex Figure Copy & Delayed Recall; MINT; Category Fluency; Phonemic Fluency
	Lin et al.	TV	Global cognition, Episodic memory	Short Portable Mental Status Questionnaire (SPMSQ); Immediate Word Recall
	Moreira et al.	TV, computer, tablet, smartphone	Memory, language, executive function	The Battery of Neuropsychological Tests CERAD; Trail Making Part B; Tests of semantic verbal fluency (flora category) & phonemic fluency
	Zhang et al.	Computer	Processing speed, reasoning, vocabulary/crystallized intelligence, working memory, executive function, subjective memory	Digit Symbol Substitution; Letter Set Test; Shipley Institute of Living Scale, Stroop Color Name Test; Trail Making Test A & B; Perception of Memory Functioning
	Zhou et al.	TV, computer/tablet	Memory (immediate recall)	Immediate Word Recall
2023	Hantke et al.	Computer	Cognitive activity/executive function (proxy); Global cognition	Mini-Mental State Examination (MMSE)
	Shuai et al.	TV, Cell phone, Computer	Global cognition	Mini-Mental State Examination (MMSE)
	Stringer et al.	Computer	Global cognition, Attention, Processing speed, Episodic memory, Executive function	Addenbrooke's Cognitive Examination; Trail Making Test Part A & B; Free and Cued Selective Reminding Test; The Doors and People Test; Digit Span Backward Test; Color-Word Interference Test (modified Stroop); Deary-Liewald Reaction Time Task

a Although not assessing cognitive function, this study includes social cognitive constructs (e.g. attitudes, modeling) as behavioral predictors of screen use. These constructs reflect psychological processes but are distinct from neurocognitive domain.

The purpose and interactivity of screen use varied across studies. Television was generally used passively for entertainment, whereas computers and smartphones were associated with more active tasks such as emailing, word processing, cognitive games, or web browsing. Several studies emphasized occupational screen use,^16,19,22^ while others focused on recreational or casual use. These variations underscore the importance of distinguishing screen type, purpose, and cognitive demand to understand their relationship to cognitive function.

Additionally, three studies^16,19,20^ integrated screen time within broader sedentary behavior patterns, recognizing that screen use may be one part of a larger constellation of health-relevant behaviors. These studies suggest that cognitive outcomes may be shaped not only by screen use itself but also by its intersection with physical activity, sleep, and other lifestyle factors.

#### Cognitive domain assessed

The findings show that most studies employ various assessments or battery tests to assess cognitive function in midlife and older adults. The cognitive domains considered in these studies are briefly presented in Table 1. The primary domains evaluated included global cognition, memory, executive function, attention, processing speed, language, and visuospatial abilities. While several studies used multi-domain neuropsychological batteries,^13,18,19,22,24,25,27,28^ others focused on specific cognitive domains.^14–17,20,21,26^

The following section summarizes key cognitive outcomes reported across the reviewed studies, beginning with general measures of cognitive impairment and then addressing findings within specific cognitive domains.

### Summary of the studies’ results

#### Cognitive impairment and risk of decline

Eight studies reported outcomes related to global cognitive functioning. These were commonly assessed using tools such as the Mini-Mental State Examination (MMSE),^14,20^ the Montreal Cognitive Assessment (MoCA),^18^ and the Short Portable Mental Status Questionnaire.^21^ Greater home computer use, including more sessions and earlier start times, was associated with higher performance across global and domain-specific cognitive tasks.^13,27^ Similarly, Kurita et al.^22^ reported that baseline computer users showed a reduced adjusted odds ratio for cognitive decline over 4 years. In contrast, Shuai et al.^20^ and Lin et al.^21^ reported mixed outcomes for television viewing, with some protective associations and others suggesting risk for impairment depending on the cognitive baseline and activity type.

Three studies directly addressed the risk of mild cognitive impairment (MCI) or cognitive decline. Community-dwelling older adults who used computers had significantly lower odds of cognitive decline after four years.^13,20,23^ Furthermore, computer use in midlife and/or late life was associated with reduced risk of developing MCI.^13,23^ Conversely, Hantke et al.^14^ reported that individuals with more severe Alzheimer's disease pathology postmortem (i.e. higher Braak NFT staging) showed significantly lower digital biomarker activity, including less computer use, suggesting that reductions in digital engagement may also reflect early neuropathological changes.

#### Domain-specific cognitive outcomes

##### Memory

Memory was assessed in 12 studies, primarily using immediate and delayed word recall tasks. Increased computer use, including gaming, word processing, and browsing, was positively associated with memory outcomes.^13,16,19,22^ Moreira et al.^19^ also identified positive associations between occupational screen time and memory performance, particularly among women. Similarly, Kurita et al.^22^ found that computer users had better recall scores over time. In contrast, Zhou et al.^16^ found that passive screen use (TV and tablet) was associated with fewer recalled words. Fancourt & Steptoe^28^ reported declines in verbal memory for individuals watching over 3.5 h of television daily. Tuteja et al.^18^ further suggested that prolonged smartphone use might contribute to hippocampal-related memory deficits among patients with Parkinson's disease.

##### Executive function and attention

Eleven studies examined executive function or attention. Tests included Trail Making Test Part B,^13,19,22–25,27^ Stroop tasks,^13,22,24,25,27^ and Symbol Digit Substitution.^22,23,25^ Time spent on word-processing applications and web browsing was positively correlated with executive function and attention.^16,19,22^ Longer computer use was associated with better task-switching and Stroop performance, highlighting potential benefits for cognitive flexibility.^13,24,25^ Moreover, screen time, mainly through occupational engagement, was positively associated with executive function, especially in men.^19^

##### Processing speed

Six studies evaluated processing speed using tools such as the Digit Symbol Substitution Test and Trail Making Test Part A. Findings were mixed. Bernstein et al.^13,24^ found that higher computer use was related to better scores on processing speed measures, whereas Zhang et al.^25^ reported no significant improvements following casual computer use over one year. The authors suggest that light, non-goal-directed screen use may not offer sufficient cognitive stimulation to enhance processing speed. Furthermore, Moreira et al.^19^ found an association between screen time and processing speed, particularly in men.

##### Language

Language outcomes were assessed in eight studies using tests such as verbal analogies,^26^ semantic and phonemic fluency,^19,28^ and the Naming Test.^13,18,23,24,28^ Several studies reported positive associations between language function and screen use, particularly with work-related or cognitively active technology use. Frequent use of word-processing and browsing applications was associated with better language performance. Moreira et al.^19^ supported this finding in a large sample. In contrast, prolonged passive television watching (e.g. >3.5 h/day) was associated with reduced semantic fluency.^19,28^

##### Visuospatial abilities

Six studies measured visuospatial function, often as part of broader neuropsychological batteries using the Benson Complex Figure, Trails A/B, or MoCA subtests. Higher computer use was associated with better visuospatial outcomes.^13,18,23^ Tuteja et al.^18^ included visuospatial performance among Parkinson's patients and noted a decline with greater mobile phone use. Krell-Roesch et al.^23^ also included visuospatial measures in their multimodal MCI classification, suggesting domain-wide cognitive engagement through screen-related activities.

#### Psychological correlates of cognition

While not directly assessing cognitive performance, Mackenbach et al.^15^ examined cognitive attitudes and modeling behaviors as psychosocial correlates of screen-based sedentary behavior. These attitudinal constructs, including perceived norms and efficacy beliefs, influence participation in cognitively engaging screen use and may moderate its effects. Positive attitudes can enhance motivation to engage in screen-based learning or cognitive activities, while negative views may reinforce sedentary habits without stimulation.

#### Active vs. passive screen time

The cognitive impact of screen use appears closely linked to the level of engagement, with growing evidence distinguishing cognitively active from passive digital behaviors. Active screen use, such as computer-based tasks involving word processing, web browsing, or cognitive games, has been consistently associated with better outcomes across domains, including global cognition, executive function, language, and memory. These associations are supported by performance on comprehensive neuropsychological batteries in studies like Bernstein et al.^13,24^ Then, Kurita et al.^22^ found that regular computer use predicted lower odds of decline across memory, attention, executive function, and processing speed. Similarly, Krell-Roesch et al.^23^ reported reduced MCI risk linked to computer use, regardless of life stage, reinforcing the cognitive stimulation hypothesis. Zhou et al.^16^ and Shuai et al.^20^ further emphasized that cognitively engaging sedentary behaviors were positively associated with cognitive performance, particularly in immediate word recall and MMSE-based global cognition assessments.

In contrast, passive screen time, such as prolonged television watching, was associated with poorer outcomes. Adults who watched TV for more than 3.5 h per day experienced declines in verbal memory and semantic fluency.^28^ Similarly, Zhang et al.^25^ found no improvements in cognitive domains like processing speed or memory among older adults engaged in casual computer use, suggesting that low-intensity engagement fails to yield cognitive benefits. Furthermore, extended mobile phone use in Parkinson's patients was linked to earlier symptom onset, potentially due to neural impacts such as hippocampal disruption.^18^

#### Moderators and contextual factors

A range of moderating factors, including education, physical activity, social engagement, sleep, and individual characteristics, influenced the cognitive effects of screen use. Education emerged as a key moderator. Lower education was linked to greater passive screen use, particularly television, while higher education was associated with more mentally active screen behaviors, and better cognitive outcomes.^15,16,19,20,26^

Social and environmental factors also played a role. Interactive screen-based activities, such as digital games, were associated with better cognitive outcomes, likely due to the combination of mental stimulation and social interaction.^19,20^ Individuals residing in urban areas tended to engage more frequently with screens, which was also associated with better cognitive performance.^20^

Physical activity further acted as a protective factor. Higher activity levels were associated with reduced cognitive risk from sedentary screen behavior.^19,20^ In this context, emerging technologies like virtual reality (VR) offer promising opportunities for combining physical movement with cognitive stimulation.

Sleep quality further moderated outcomes. Greater computer use, combined with more prolonged and earlier sleep, was linked to better cognitive functioning.^18,24^ It suggests that screen use within healthy routines may be beneficial.

Lastly, individual factors such as age, mental health, and gait speed influenced the benefits of screen use. Calhoun and Lee^26^ found that computer use was more beneficial among older adults who were younger, more educated, and physically and mentally healthier. These findings highlight that the effects of screen time on cognition are not universal but rather depend on broader lifestyle, health, and social contexts.

## Discussion

This scoping review examined how screen use is associated with cognitive function in midlife and older adults. The evidence highlights a complex and context-dependent relationship, with both positive and negative associations emerging across cognitive domains. Studies investigating cognitively engaging digital activities, such as email, word processing, cognitive games, and web browsing, suggest potential cognitive benefits, particularly in domains such as memory, attention, and executive function.^13,16,17,19,20,22,24,26^ These findings support the cognitive stimulation hypothesis, which posits that mentally active engagement, including through digital devices, can help preserve function in aging populations.^4–7^ These benefits were most consistently observed in memory, attention, and executive function domains, whereas outcomes for processing speed and visuospatial abilities were more variable.

In contrast, studies focusing on passive screen activities, such as prolonged television viewing, report associations with poorer outcomes, including diminished verbal memory and global cognition.^18,21,28,29^ Furthermore, some longitudinal findings indicate null or mixed effects, with factors such as screen content, usage duration, and user characteristics (e.g. age, education, baseline cognition) likely moderating observed outcomes.^15,17,19,20,23,25,26^

This pattern aligns with the distinction between active and passive digital behaviors, where active engagement (e.g. browsing, games, emailing) was linked to more favorable cognitive outcomes, while passive viewing (e.g. prolonged TV watching) showed greater risk for cognitive decline. These mixed findings underscore the importance of differentiating not only screen type (e.g. TV, computer, smartphone) but also the cognitive demands, purpose, and context of screen-based activities. While active screen use may reinforce cognitive skills and promote engagement, excessive or low-stimulation use may displace more enriching behaviors and contribute to decline.

Attitudinal factors were also explored, with one study finding that beliefs about mental activity and aging may shape screen behaviors in ways that interact with cognition.^15^ Recent literature further supports the view that individuals’ perceptions of their digital competency, digital health literacy, and prior exposure to technology can significantly influence both the frequency and type of screen use, with downstream effects on cognitive health.^30,31^ Notably, the heterogeneity in findings and study designs limits causal inference, reinforcing the need for future research using longitudinal or experimental approaches that isolate key mechanisms.

This review also underscored the moderating influence of sociodemographic, behavioral, and psychological factors, such as education, physical activity, sleep quality, urban living, and individual attitudes, on the relationship between screen use and cognitive outcomes. Lower education was linked to more passive television use, whereas higher education was associated with cognitively stimulating screen behaviors.^15,16,19,20,26^ Screen-based leisure activities, particularly games, offered dual cognitive and social benefits, especially among urban dwellers.^20^ Physical activity buffered the cognitive risks of sedentary screen use, while emerging technologies like VR and AR present integrated pathways for simultaneous cognitive and physical engagement. Sleep quality also moderated outcomes; longer duration, earlier bedtimes, and higher computer use were associated with better cognitive performance.^18,24^ Lastly, individual characteristics such as younger age, better mental health, and faster gait speed enhanced the cognitive benefits of screen use,^26^ reinforcing that digital engagement is embedded within broader developmental, health, and contextual frameworks.

This review highlights that screen use is neither inherently beneficial nor harmful to cognitive function in aging populations. Instead, its cognitive impact depends on multiple interacting factors, including device type, activity complexity, individual user characteristics, and broader lifestyle contexts. This suggests that digital engagement must be interpreted within developmental and contextual frameworks reflecting diverse aging trajectories, technological access, and digital learning opportunities.

While this review highlights several promising and concerning trends in how screen use may influence cognition in midlife and older adults, the overall evidence base remains limited by methodological inconsistencies. Many included studies relied on self-reported screen use, introducing potential biases such as recall error or social desirability. Sample sizes ranged widely, and several studies lacked detailed demographic or contextual information necessary to assess confounding influences. The quality of cognitive assessments also varied, with some studies using robust neuropsychological batteries while others relied on brief global screens.

Future research on digital engagement and cognitive aging would benefit from incorporating tools such as ecological momentary assessment (EMA), passive digital tracking, and objective biomarkers to improve real-time measurement precision. Utilizing these tools could improve consistency across studies and help clarify currently mixed findings. This, in turn, would support the development of more rigorous and inclusive research on digital engagement and cognitive aging.

## Limitations and strengths

This scoping review has several limitations. First, the literature search was limited to two databases and restricted to studies published between 2018 and 2023. While this timeframe captured recent evidence during a period of rapid digital adoption, it may have excluded relevant studies from other databases. Second, although general terms like “screen use” and “digital screen exposure” were included, device-specific terms such as “tablet” were not explicitly searched. While two tablet-inclusive studies were identified, this omission may have limited device representation. Third, the geographic distribution was skewed toward high-income Western countries, constraining generalizability and underscoring the need for more inclusive global research.

Despite these limitations, the review offers several strengths. It systematically synthesizes recent evidence on screen use and cognitive outcomes in midlife and older adults, a growing yet underrepresented population in digital health research. By including diverse screen types (TV, computers, smartphones, tablets) and cognitive domains (e.g. memory, attention, executive function), the review provides an integrative perspective. Charting study characteristics and cognitive measures contributes to a conceptual framework distinguishing cognitively active from passive digital behaviors. Finally, the review identifies methodological gaps and provides direction for future research on digital aging and cognition.

## Conclusion

This scoping review reveals that screen use in midlife and older adulthood has both potential benefits and risks for cognitive function, depending on the type of activity, device, and context. Active, cognitively engaging screen use may support domains like memory and executive function, while passive behaviors such as prolonged television viewing are linked to poorer outcomes. However, variability in study designs and limited global representation constrain generalizability. Future research should address these gaps through diverse sampling, standardized measures, and a clearer distinction between screen types and uses to inform cognitive health strategies in aging populations better.

## Appendix A:

**Table A1.** Summary of the included studies.

Year	Author(s)	Study location	Study type	Purpose/aims	Participants (sample size)	Screen use	Cognitive performance	Findings/discussion
2019	Calhoun & Lee	USA	Quantitative: Longitudinal cohort study using secondary analysis of nationally representative HRS data (multistage probability sampling)	The study analyzed the effect of computer usage on the cognitive capabilities of older adult (50 and older)	Older adult aged ≥50 (*N* = 5259)	Computer use per week (hours)	Cognitive ability: Verbal series scores from 2014 core survey (verbal analogies test)	Increased use of computers is associated with improved cognitive capability (when controlling for age, gender, and years of education)
2019	Couth et al.	UK	Qualitative: Delphi-style expert consensus method via two workshops and a follow-up survey; convenience sampling of six cognitive and clinical neuroscience experts from UK universities and the National Health Service	The study involves panel of expert to gain a consensus for computer use behaviors that would be a strong indication of decline on cognitive function	Expert from clinical and cognitive neurosciences (*N* = 6)	Computer use (desktop/laptop)	Cognitive ability: Memory, executive function, language, perception, and action	This resulted in a list of 21 computer-use behaviors that the majority of experts agreed would offer a ‘strong indication’ of decline in a specific cognitive function, across Memory, Executive function, Language and Perception and Action domains
2019	Fancourt & Steptoe	England	Quantitative: Secondary longitudinal analysis of a nationally representative cohort (ELSA), using stratified probability sampling	The study aimed to explore whether television viewing behavior in adults aged 50 or mor are associated with a decline in cognition	Adults aged ≥ 50 from the English longitudinal study of aging (*N* = 3662)	TV watching (hours per day)	Cognitive decline: Measure through verbal memory and semantic fluency	Watching television for more than 3.5 h per day is associated with a decline in verbal memory.
2019	Krell-Roesch et al.	USA	Quantitative: Population-based prospective cohort study (Mayo Clinic Study of Aging), with purposive sampling applied retrospectively based on baseline cognitive status and activity engagement	The study investigates whether timing, number, and frequency of mentally stimulating activities in midlife and late life are associated with the risk of incident mild cognitive impairment	Adults aged ≥ 70 years were cognitively unimpaired at baseline.	Computer use	Mild cognitive impairment (MCI): Determined by panel of expert according to neurological examination, risk factor assessment, and neuropsychological testing. Neurological examination: neurological history review and administration of short test of mental status Risk factors assessment: Clinical Dementia Rating Scale (CDR) Neuropsychological testing: assessing 4 domains of cognition (memory, language, visuospatial skills, attention/executive function)	Increased use of computers is associated with improved cognitive capability (when controlling for age, gender, and years of education)
2019	Mackenbach et al.	Belgium	Quantitative: Cross-sectional study using random sampling from a population registry (non-stratified sampling)	The study examines whether social cognitive, home environmental, and health-related factors contributed to socioeconomic difference in television-related sitting time	Adult (25–60 years) (*N* = 301) and Older adult (≥65 years) (*N* = 258)	Television-related sitting time	Social cognitive: Attitude, modelling, self-efficacy, social norm, norm, social support, and modelling	Social cognitive variables explained large part of educational inequalities leading to more television-related sitting time
2020	Nori et al.	Italy	Quantitative: Cross-sectional experimental study using convenience sampling	The study analyzes the relationship between web-searching navigation and age	*N* = 18 young adults (age 18–30) *N* = 18 young-old adults (age 60–75)	Computer use: Frequency, proficiency, and modalities of technological device use: games, word processor, calculation and statistical software, emails, programing, educational programs, web browsing, and chat Additional question: smartphone/tablet usage, most used browser, use of and time spent on social media vs general web searching	Cognitive function: Cognitive impairments are controlled. Measured by MMSE (rapid assessment of mental state of individual identifying the presence of possible cognitive deterioration) and Kaufman brief intelligence test (general measure of cognitive function includes verbal knowledge, riddles, and matrices)	Age did not influence web-searching behavior in users with normal expertise and intelligence. Older participants spend more time in web-searching but have the same accuracy in solving the problem
2021	Bernstein et al.	USA	Quantitative: observational cross-sectional study used convenience sampling	The study examines whether routine home computer use could effectively discriminate between older adults with and without MCI	N total = 60 Community-dwelling older adults (*n* = 39 cognitively healthy, *n* = 21 with MCI)	Computer use (measured by worktime computer use monitoring software, then categorized into 34 categories)	Cognitive impairment (MCI) Neuropsychological battery: these measures cover several cognitive domains, including attention, processing speed, memory, language, executive function, and visuospatial construction.	Cognitively health participants spent mor time using the computer, had greater number of computer sessions, and had earlier mean time of first daily computer session. Better cognitive, particularly in memory and language are associated with frequency of browser, word processing, search, and game application use.
2021	Kurita et al.	Japan	Quantitative: Prospective longitudinal cohort study using data from the National Center for Geriatrics and Gerontology; convenience sampling of community-dwelling older adults, with stratified analyses by demographic and functional characteristics	The study examines the association between computer use and cognitive decline among community-dwelling older adults	Older adult (cognitively intact at wave 1) and then followed through 4 years later (completed sample (*N* = 2010)	Computer use (yes or no question)	Cognitive decline: Measured by the National Center for Geriatrics and Gerontology-functional Assessment Tool (NCGG-FAT) 4 Cognitive domains: immediate recognition, delayed recall, attention, and executive function Scoring bellow threshold in at least one of four neuropsychological tests at wave 2	Computer use is longitudinally associated with protected cognitive function
2021	Tuteja et al.	India	Quantitative: Cross-sectional observational study using convenience sampling	The study examines the changes in cognitive impairment, sleep pattern, visuospatial ability in Parkinson's disease and its association with smartphone use	Parkinson's patients 40–60 years) Group I: ≥ 60 (*N* = 27) and Group II: 40–60 years (*N* = 21)	Smartphone use: The average time spent on calls or messaging, Number of calls/days, Mobile phone usage and timespan to use the mobile phone	Cognitive ability: Montreal cognitive assessment (MOCA) a 30 points test includes important cognitive parameter: alternative trial making, visuospatial constructional skills, naming, memory, attention, sentence repetition, verbal fluency, abstraction, delayed recall, and orientation. Score less than 26 is considered as mild impairment in cognitive ability	The usage of mobile phone might contribute to the early onset of the severity of symptoms associated with Parkinson's disease
2022	Bernstein et al.	USA	Quantitative: Observational cohort study (short-term longitudinal) using purposive sampling from existing research registries and healthcare systems	The study examined whether a comparatively brief data may yield similar diagnostic information	Older adults aged ≥60 without dementia at the baseline (*N* = 91)	Computer use (the number and duration of computer sessions), wearable sleep tracker, instrumented pillbox	Cognitive ability: Through clinical assessment procedures involving several assessments Standardized battery of health and function questionnaires Neuropsychological battery Neuropsychological measure These measures cover several cognitive domains, including attention, processing speed, memory, language, executive function, and visuospatial construction.	Greater computer use associated with better cognition
2022	Lin et al.,	Taiwan	Quantitative: Longitudinal cohort study with stratified multistage probability sampling	The study aimed to identify heterogeneous cognition, depression, and life satisfaction trajectory groups, and to examine the independent contributions of watching television and reading to these trajectories among middle-aged and older adults	Adults aged 45 years and older (*N* = 4440), with subgroup trajectories stratified by age groups (e.g. 45–64 = middle-aged; 65+ = older adults).	Television watching	Cognitive ability: Global Cognition: measured using Short Portable Mental Status Questionnaire (SPMSQ) Episodic memory: immediate word recall	Watching television lower the odds of having lower global cognitive function
2022	Moreira et al.	Brazil	Quantitative: Cross-sectional study using convenience sampling from the ELSA-Brasil cohort	The study aimed to verify the association between sedentary behavior and performance on cognitive function test in middle-aged and older adults	ELSA-Brazil Sample *N* = 6505 adults aged ≥ 55	TV watching, Computer, Tablet, Smartphone (accumulative of time spent)	Cognitive performance: Memory: immediate and delayed memory, and also ability to remember & distinguish correct words from set of distractors Language: semantic verbal fluency, phonemic fluency Executive function: attention, concentration, psychomotor speed, and mental flexibility	SB was found to be related to better performance in memory, language, and executive function among men, while related to memory among women. Screen time was found to be associated with executive function. Among women, occupational screen time were positively associated with memory performance.
2022	Zhang et al.	USA	Quantitative: Secondary analysis of longitudinal Randomized controlled trial (RCT) data	The study examined whether computer use improves cognition in older adult with no prior experience with computer usage	Older adult (age ≥ 65) with no previous computer experience, and at risk for social isolation. Experiment: technology-based social support (*N* = 150) and Control: non-technology-based social support through paper blinder (*N* = 150)	Computer use, The number of use PRIMS system	Cognitive improvement measured through a cognitive battery measurement, including processing speed, reasoning, crystallized intelligence, working memory, and executive function. A measure of everyday memory, and memory functioning: frequency of forgetting, seriousness of forgetting, and retrospective functioning.	Casual computer use does not prove enough cognitive stimulation to improve cognition in late adulthood.
2022	Zhou et al.	USA	Quantitative: Cross-sectional; nationally representative survey data using stratified multistage probability sampling	The study identifies distinct subgroups of older adult with six domain s of sedentary behavior and compare them according to health-related outcomes	Older adults aged ≥80 years (*N* = 852)	Computer use, tablet, TV watching (time spent)	Cognitive ability: Cognitive function: using immediate word recall Self-report on chronic condition such as dementia. Number of difficulties in activities of daily living Number of problems limiting activities, such as balance coordination problems	High-mentally active group were associated with higher cognitive function
2023	Hantke et al.	USA	Quantitative: Cross-sectional analysis of a longitudinally monitored convenience sample	The study aims to perform an exploratory examination of possible associations between DBs and AD neuropathology in an initially cognitively intact community-based cohort	Older adults aged ≥65 years, living independently, having average health for age, and followed until death	Computer use: through monitoring software installed in the computer	Cognitive ability: Clinical Dementia Rating (CDR) Digital Biomarker such as computer use, walking speed, time spent outside the home, and time spent in bed Evaluation on postmortem brains using NFTS and NP Technology	These DBs were found to be linked with Alzheimer's disease (AD) signs in the brain, such as tangles and plaques, in older adults who were initially cognitively normal. People with more severe plaques had lower DBs for walking speed and time in bed, suggesting these DBs might be sensitive to amyloid buildup in the brain. People with more severe plaques had lower DBs for walking speed and time in bed, suggesting these DBs might be sensitive to amyloid buildup in the brain
2023	Shuai et al.	China	Quantitative: Longitudinal cohort study using multistage purposive sampling, with both baseline cross-sectional and 2-year prospective analyses	To explore the relationship between forms of sedentary behavior with cognitive function among community-dwelling older adults in Chinese	Participants aged 60 years and older *N* = 5356 at a baseline *N* = 956 at 2-year follow-up	TV, Cell phone, Computer (Total hours of sedentary duration)	Cognitive function: Measured through Mental state (Mini Mental State Examination (MMSE)): tool for evaluating cognitive function including memory, orientation, language, attention, and computation. Higher scores indicate better cognition.\ Mild Cognitive Impairment (MCI) is classified by having an MMSE score lower than 17 (0 years of education), 20 (elementary school), and 24 (at least secondary school).	The impact of forms of sedentary behavior on cognitive function was found. Participants who reported longer screen-watching sedentary duration had higher MMSE scores and lower likelihoods of MCI
2023	Stringer et al.	UK	Quantitative: Longitudinal study using purposive sampling	The study explored whether computer-use behaviors recorded. during routine home computer-use (1) could discriminate between individuals with subjective cognitive decline (SCD) and individuals with mild cognitive impairment (MCI); ii) were associated with cognitive. and functional scores; and iii) changed over time.	Older adults aged ≥65 years (*N* = 32): Individuals with subjective cognitive decline (*n* = 18) and mild cognitive decline (*n* = 14)	Computer use: through continuous recording of specific computer activities	Cognitive ability: Battery of cognitive and functional assessment: visual and verbal memory elements	Individuals with MCI had significantly slower keystroke speed and spent less time on the computer than individuals with SCD. More time spent on the computer was associated with better task switching abilities. Faster keystroke speed was associated with better visual attention, recall, recognition, task inhibition, and task switching. No significant change in computer-use behavior was detected over the study period.
